# Changes in subchondral bone morphology with osteoarthritis in the ankle

**DOI:** 10.1371/journal.pone.0290914

**Published:** 2024-06-18

**Authors:** Lekha Koria, Mark Farndon, Elena Jones, Marlène Mengoni, Claire Brockett

**Affiliations:** 1 Institute of Medical and Biological Engineering, School of Mechanical Engineering, University of Leeds, Leeds, United Kingdom; 2 Harrogate and District NHS Foundation Trust, Harrogate, United Kingdom; 3 Leeds Institute of Rheumatic and Musculoskeletal Medicine, School of Medicine, University of Leeds, Leeds, United Kingdom; University of Life Sciences in Lublin, POLAND

## Abstract

Significant alterations to subchondral trabecular bone microarchitecture are observed in late-stage osteoarthritis (OA). However, detailed investigation of these changes to bone in the ankle are under-reported. This study aimed to fully characterise the trabecular morphology in OA ankle bone specimens compared to non-diseased (ND) controls using both standard and individual-trabecular segmentation-based (ITS) analyses. Ten ND tibial bone specimens were extracted from three cadaveric ankles, as well as five OA bone specimens from patients undergoing total ankle arthroplasty surgery. Each specimen was scanned using microcomputed tomography from which a 4 mm cuboidal volume was extracted for analysis. Morphological parameters for the subchondral trabecular bone were measured using BoneJ (NIH ImageJ) and 3D ITS for whole volumes and at each depth level in 1 mm increments. The results show an overall increase in bone volume fraction (p<0.01) and trabecular thickness (p<0.001) with OA, with a decrease in anisotropy (p<0.05). ITS analysis showed OA bone was composed of more rod-like trabeculae and plate-like trabeculae compared to ND bone. Numerous properties were depth dependent, but the results demonstrated that towards the subchondral bone plate, both rod- and plate-like trabeculae were thicker, rods were longer and plates had increased surface area. Overall, this study has verified key microstructural alterations to ankle subchondral bone that are found in other OA lower-limb joints. Depth-based analysis has highlighted differences of interest for further evaluation into the remodelling mechanisms that occur with OA, which is critical to understanding the role of subchondral bone microarchitecture in the progression of the disease.

## 1. Introduction

Ankle osteoarthritis (OA) is a debilitating condition affecting the talocrural joint. Ankle OA predominantly develops post-traumatically, with symptoms presenting many years after the initial sprain or trauma [1]. An increasingly younger population of patients are therefore seeking treatment for ankle OA [2–5], much earlier than typically required in other lower limb joints [6]. This presents a clinical challenge to allow these young patients to maintain their active lifestyles with the presence of ankle OA. The main conservative treatments most commonly used today, aimed at managing pain and restoring the physiological properties of the joints are paracetamol, non-steroidal anti-inflammatory drugs (NSAIDs), intra-articular infiltration (corticosteroids, hyaluronic acid and platelet-rich plasma), physiotherapy and instrumental physical therapy [7–10]. Total ankle arthroplasty aims to restore mobility to a late-stage OA joint, but requires resection of bone from the articulating surfaces of the distal tibia and talus [11]. However, it can be difficult to stabilise the implant due to poor bone quality [12], and therefore many implants suffer from failures modes such as stress- shielding, loosening and subsidence [13, 14]. Compared to other lower limb joints, total ankle replacements are reported to have relatively high revision rates [15, 16].

The increased apparent stiffness reported in late-stage OA bone [17–19] is known to correlate with alterations to the microstructure that occur as a result of accelerated, irregular bone remodelling [20]. It is well known that bone density and trabecular thickness increase with OA [19–23], but also the combination and properties of the trabecular types (rod and plate) also alter [19, 23, 24]. Such changes are under-reported in the ankle joint, but fully characterising these alterations will aid in understanding the correlations between microarchitecture and apparent mechanical properties, which can be invaluable for predicting fracture risk or developing novel implant designs [17–19, 25].

Hence the aim of this study was to evaluate the changes to subchondral trabecular bone in the osteoarthritic ankle by employing both standard and individual-trabecular segmentation (ITS)-based morphological analyses. Variations to each morphological property with increasing depth from the subchondral bone plate was also evaluated.

## 2. Materials and methods

### 2.1. Samples

**Fig 1:**
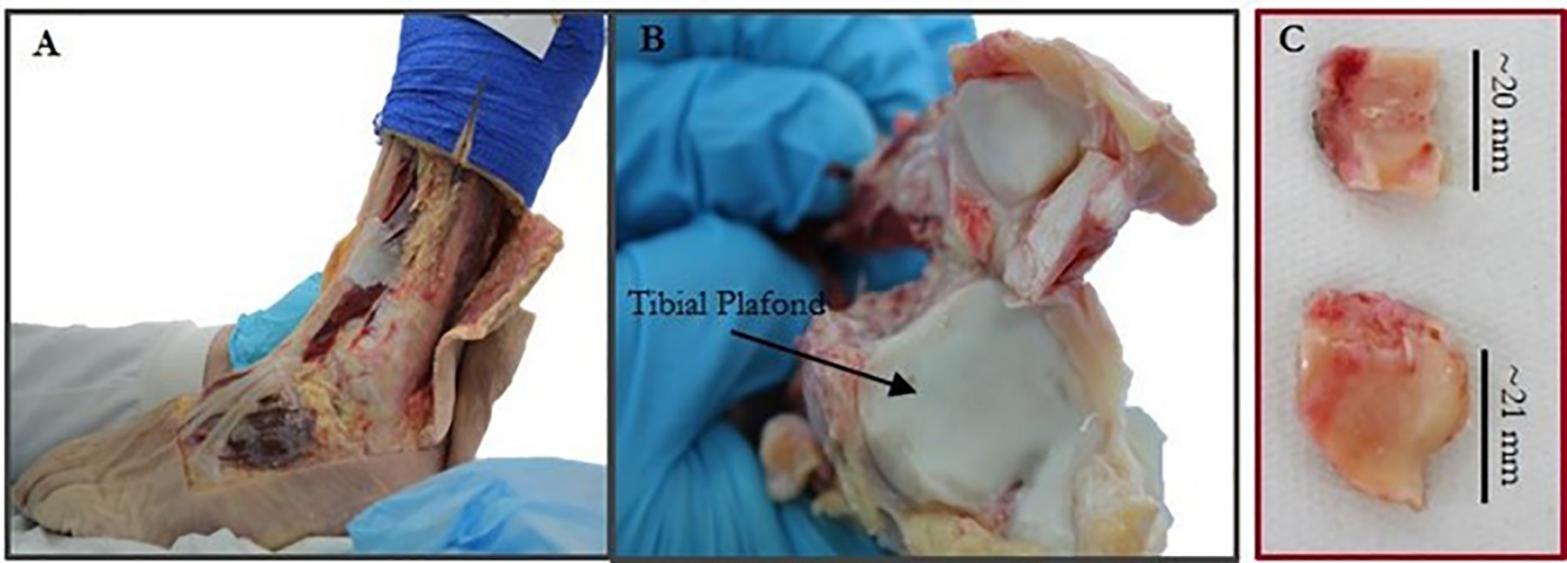
Location of an exemplary set of late-stage osteoarthritic ankle bone samples obtained from patient undergoing total ankle arthroplasty.

Non-diseased (ND), human cadaveric feet (MedCure USA) were sourced for this study, with consent acquired by the providing company (tissue accessed February to April 2019). Ethical approval was granted by the faculty research ethics committee (MEEC 15–020). Exclusion criteria for the tissue included prior lower limb trauma or surgery, or a history of diabetes. Each foot was stored at -80°C in a freezer prior to dissection, in compliance with the Human Tissue Act. Late-stage OA specimens were sourced from patients undergoing total ankle arthroplasty (Fig 1). Ethical approval was obtained from NREC National Research Ethics committee in compliance with the Helsinki Declaration of ethical principles for medical research involving hu- man subjects. All patients provided written consent for this study. Each patient underwent total ankle arthroplasty surgery, but the cause of OA (i.e. post-traumatic or not) was unknown. Tissues were accessed until March 2020.

### 2.2. Specimen preparation

#### 2.2.1. Non-diseased specimens

Each foot was thawed for at least 24 h at 4°C in a refrigerator. Prior to dissection each foot was imaged at an isotropic resolution of 82 *μ*m using a HR-pQCT scanner (XtremeCT, ScanCo Medical, Brüttisellen, Switzerland). This was performed to ensure no major undiagnosed damage or bone cysts were present.

The distal tibia was dissected from each ankle by specialist foot and ankle consultant orthopaedic surgeons (Fig 1). Surrounding soft tissues were removed with a scalpel and each bone was wrapped in tissue soaked in phosphate-buffered solution (PBS) to maintain hydration until specimen removal. Subchondral bone cores (N = 10) were removed from the tibial plafond using a hammer and tubular chisel (6.5 mm; Smith and Nephew Plc, Wat- ford, England). Cores were extracted from both medial & lateral, anterior and posterior regions; two to four cores per tibia were of sufficient quality for use in the study. The articular cartilage and subchondral bone plate (SBP) were removed using a scalpel. Each end was sanded to a flat-surface and rinsed with PBS to remove debris. The average measured specimen height was 8.46 *±* 1.13 mm. Each specimen was wrapped in PBS-soaked tissue and refrigerated at 4°C until imaging.

#### 2.2.2. Late-stage osteoarthritic specimens

Each specimen retrieved in surgery (Fig 1) was placed into saline immediately after retrieval with annotation of the specimen orientation with respect to the distal tibia. Each of the five specimens were transported on the day of retrieval to the laboratory. Specimens were then stored at -80°C in a freezer prior to imaging.

### 2.3. *μ*CT imaging

Each OA specimen and ND subchondral bone core were imaged at an isotropic resolution of 16 *μ*m (70 kVP, 114 *μ*A, 250 ms integration time) using a desktop micro-CT (*μ*CT 100, ScanCo Medical, Brüttisellen, Switzerland). Specimens were submerged in PBS during scanning to maintain hydration. The data was normalised to a 0–255 grayscale (8 bit).

A 4 mm cuboidal volume was extracted from the top centre of each ND image stack (NIH ImageJ v1.53 [26]). Similarly, a 4 mm trabecular bone volume was extracted just beneath the subchondral bone plate from each OA specimen. All image stacks were binarised using Otsu thresholding.

### 2.4. Subchondral bone analysis

**Fig 2:**
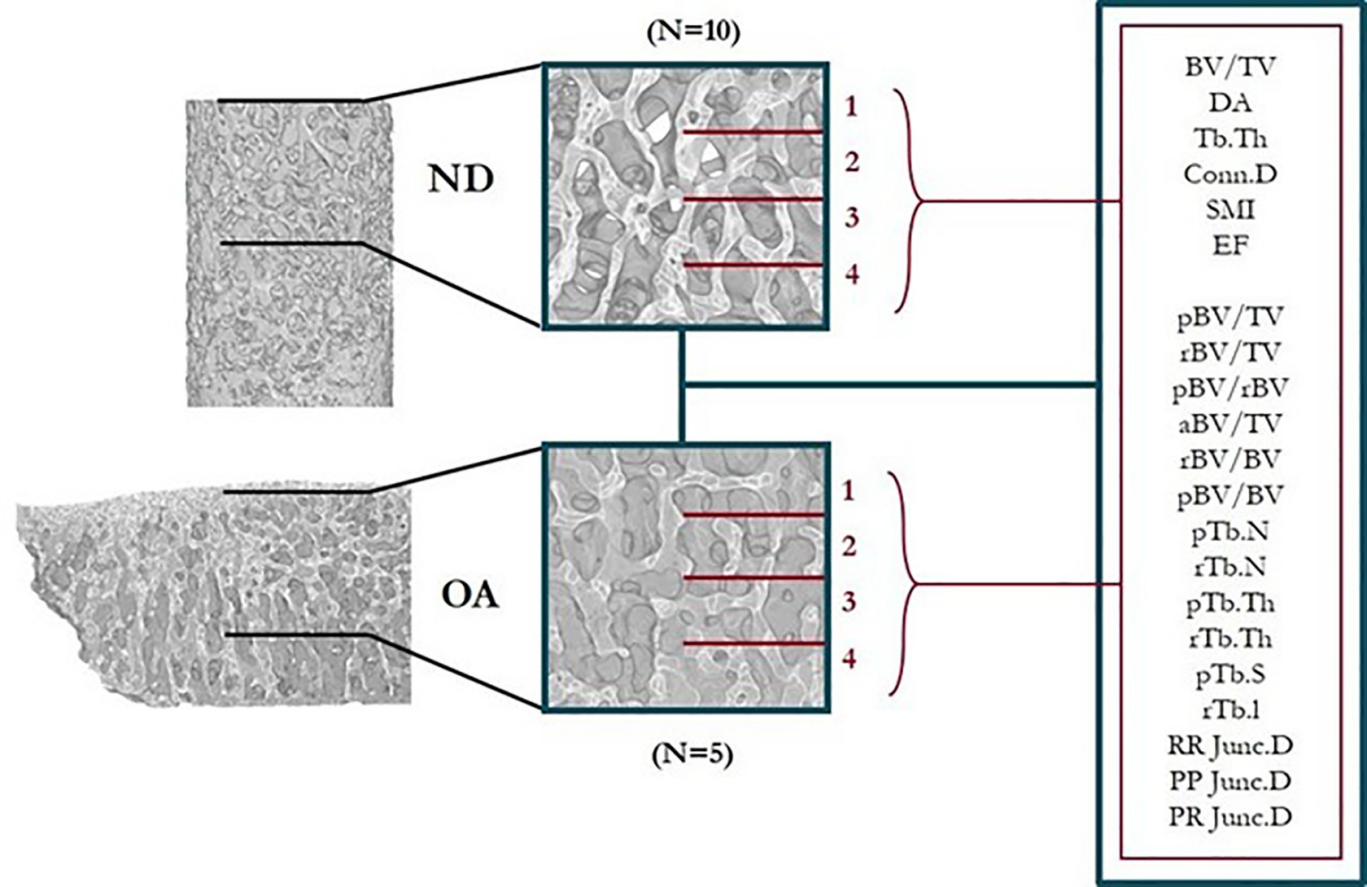
Study workflow showing exemplary cuboidal portions of ND and OA bone taken from μCT image data for full morphological evaluation. The volumes were also split into four 1 mm levels to evaluate the sensitivity of each morphological property to increasing depth from the subchondral bone plate.

Standard morphological evaluation was performed using the BoneJ (1.4.2, [27])) plugin for ImageJ within to evaluate the following properties: bone volume fraction (BV/TV); the degree of anisotropy (DA); trabecular thickness (Tb.Th, mm); connectivity density (Conn.D, mm-3); structure model index (SMI) and ellipsoid factor (EF) (Fig 2). EF results were further analysed using the Flinn peak plots, which depict the volume-weighted axis ratios of the fitted ellipsoids (a/b and b/c).

3D Individual Trabecula Segmentation (ITS) Morphological Analysis and Modelling Technique [28] was used evaluate the microstructure in more detail [29–31]. ITS-based parameters were calculated, including: plate and rod bone volume fractions (pBV/TV and rBV/TV, %); plate-to-rod ratio (pBV/rBV); axial bone volume fraction (aBV/TV); plate and rod tissue fractions (pBV/BV and rBV/BV); trabecular plate and rod numerical density (pTb.N and rTb.N, 1/mm); plate and rod thicknesses (rTb.Th and pTb.Th, mm); rod length and plate surface area (rTb.l, mm and pTb.S, mm2) and, plate-rod, rod-rod, and plate-plate junction density (PR/RR/PP Junc.D; 1/mm3).

Both standard and ITS-based properties were evaluated for all ND and OA cuboidal volumes. To evaluate sensitivity of each property with depth, each bone volume was cropped to regular 1 mm levels from the top surface, defined below:

- Level 1 = 0–1 mm
- Level 2 = 1–2 mm
- Level 3 = 2–3 mm
- Level 4 = 3–4 mm

Full morphological analyses (standard and ITS) was then performed on each volume at each depth level for all specimens (Fig 2).

### 2.5 Data analysis

The data associated with this paper are openly available from the University of Leeds research data repository [32]. The mean (AVG) and standard deviation (*±* SD) was evaluated for each morphological property. Independent samples t-test with Bonferroni correction (*α* = 0.05) was used to compare significant differences between OA and ND morphological properties. Samples were randomised during analysis and whilst some were from the same donor, the samples themselves were non-identical as they were sourced from different regions of the bone.

## 3. Results

### 3.1. Samples

A total of three ND specimens were used in this study. Tissue donors were all male (BMI: 20.61, 28.13 and 29.56 kg *m*^−2^) and were aged 43, 50 and 57 years. Similarly, a total of five OA samples were obtained for this study. Tissue donors were all male, aged 58, 65 and 68 years.

### 3.2. Standard morphological properties

**Fig 3:**
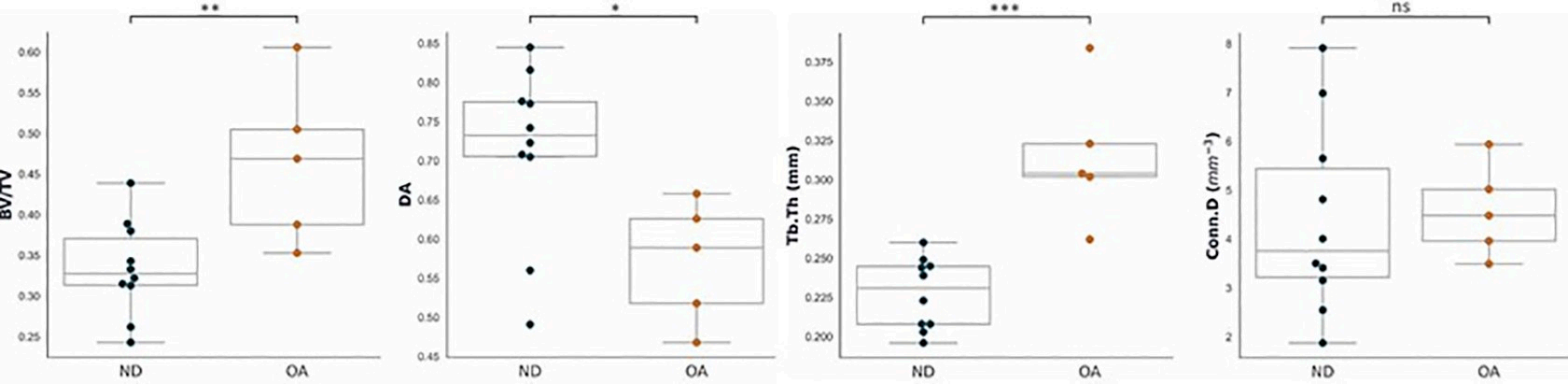
Distribution of standard morphological values for OA vs. ND groups of distal tibial specimens. The box plots represent the median and interquartile range of values for N = 5 OA specimens and N = 10 ND specimens. Significance levels are indicated using: *p<0.05, ** p<0.01, *** p<0.001, ns = non-significance.

**Fig 4:**
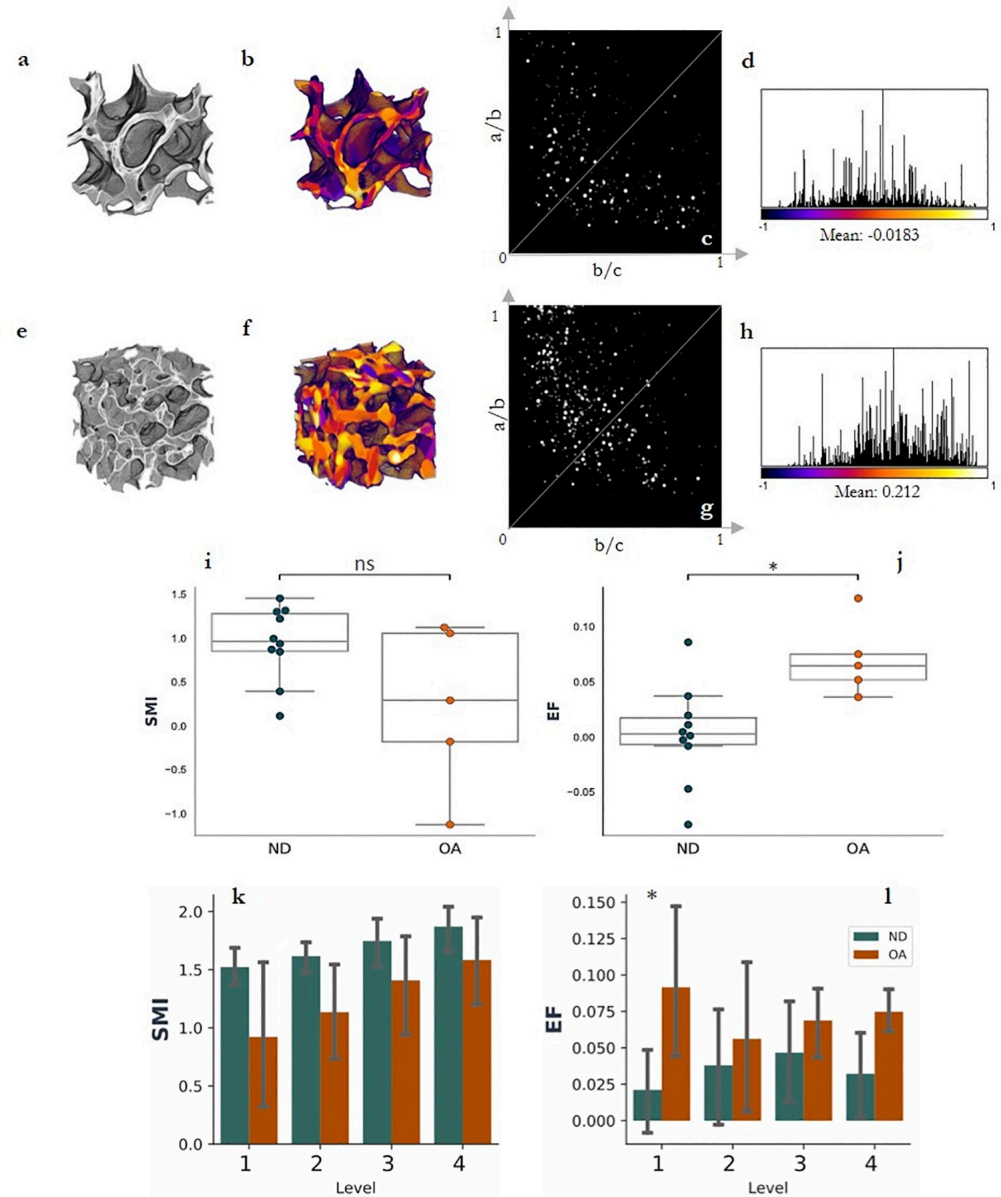
A comparison between ND (a) and OA (e) exemplary specimen subvolumes (2 mm). EF results show EF >0 (yellow-orange, f) or more rod-like trabeculae in the OA specimens. In comparison, ND specimens show EF <0 (purple-blue, b) indicating more plate-like trabeculae. Finn peak plots (c,g) show shift to of distribution toward the bottom-right for plate-dominated geometry (c) and top-left for rod-dominated geometry (g). Histograms and summary statistics (d,h) further demonstrate the shift in values towards either +1 (h; rod-dominated geometry) or -1 (d; plate-dominated geometry). The box plots (i, j represent the median and interquartile range of EF and SMI values for (N = 5) OA specimens and (N = 10) ND whole cuboidal specimens. Significance levels are indicated using: * p<0.05, ns = non-significance.

Significant increases in BV/TV (p<0.01) and Tb.Th (p<0.001), and decreases in DA (P<0.05), were observed with OA (Table 1 and Fig **3**). A significant increase in mean EF value (p<0.05) showed increased rod-like trabeculae present in OA specimens (Table 1 and Fig **4**).

**Table 1:** Mean (AVG) and standard deviation (*±* SD) values of the standard and ITS-based morphological properties from OA and ND distal tibia specimens. Statistically significant differences between both groups were calculated using an independent samples t-test with Bonferroni correction. Levels of significance are indicated using: * (p<0.05), ** (p<0.01), *** (p<0.001) and ns (p>0.05).

	OA		ND		
	AVG	*±*SD	AVG	*±*SD	p, ND vs. OA
**BV/TV**	0.464	0.100	0.334	0.058	0.0066 **
**DA**	0.572	0.078	0.714	0.110	0.0238 *
**Tb.Th (mm)**	0.315	0.045	0.228	0.023	0.0002 ***
**Conn.D (mm-3)**	4.581	0.951	4.387	1.945	ns
**EF**	0.071	0.034	0.003	0.045	0.0101 *
**SMI**	0.237	0.933	0.949	0.423	ns
**pBV/TV (%)**	42.293	9.237	31.597	5.310	0.0127 *
**rBV/TV (%)**	3.985	1.156	1.793	0.600	0.0003 ***
**pBV/rBV**	11.032	2.573	18.587	3.762	0.0015 **
**aBV/TV**	0.256	0.053	0.230	0.028	ns
**pBV/BV**	0.913	0.022	0.947	0.010	0.0009 ***
**rBV/BV**	0.087	0.022	0.053	0.010	0.0009 ***
**pTb.N (1/mm)**	3.361	0.222	3.078	0.308	ns
**rTb.N (1/mm)**	2.424	0.214	1.928	0.209	0.0009 ***
**pTb.Th (mm)**	0.148	0.002	0.139	0.006	0.0102 *
**rTb.Th (mm)**	0.090	0.003	0.090	0.002	ns
**pTb.S (mm2)**	0.075	0.003	0.079	0.012	ns
**rTb.l (mm)**	0.355	0.029	0.318	0.014	0.0047 **
**RR Junc.D (1/mm3)**	1.816	0.904	1.277	0.480	ns
**PR Junc.D (1/mm3)**	30.497	7.752	17.541	5.393	0.0022 **
**PP Junc.D (1/mm3)**	30.316	6.607	22.703	7.124	ns

To minimise the number of data points in the Flinn peak plots (Fig 4C and 4G) and histograms (Fig 4D and 4H), smaller cuboidal subvolumes (2 mm) were extracted from two exemplary specimens (Fig 4), which showed an apparent increase of rod-like trabeculae in the OA group.

Comparatively, a decrease in SMI was observed in the OA group, indicating an increased presence of plate-like trabeculae in OA specimens, though this was non-significant (p>0.05).

### 3.3. ITS-based morphological properties

**Fig 5:**
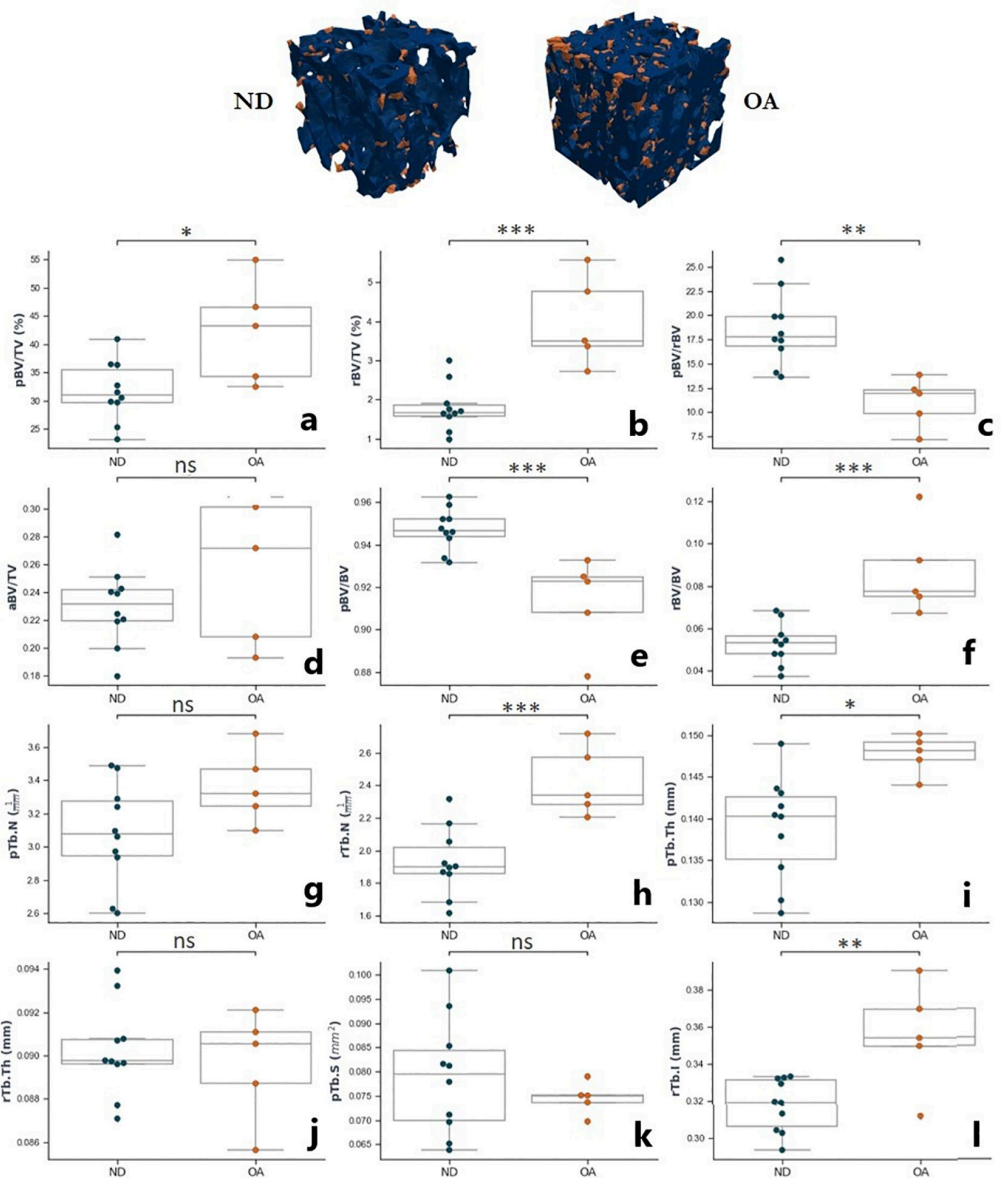
Distribution of ITS-based morphological values for OA vs. ND groups for specimens from the distal tibia. Two exemplary volumes show the increasing in rod-like trabeculae (orange) compared to plate-like trabeculae (blue) in OA specimens. The box plots represent the median and interquartile range of values for OA specimens (N = 5) and ND specimens (N = 10). Levels of significance indicated using: * p<0.05, ** p<0.01, *** p<0.001, ns = no significance.

The ITS-based morphological results showed significant increases to rBV/TV (p<0.001) and rBV/BV (p<0.001) in the OA group compared to controls (Fig 5B and 5F). In the OA group, there was increased pTb.Th (p<0.05) and a reduction in pBV/BV (p<0.001) resulting in an overall decrease in pBV/rBV (p>0.01) (Fig 5I, 5E and 5C respectively). Whilst standard morphological analysis showed significant decreases in DA with OA (p<0.05), aBV/TV was not significantly different between the two groups (Fig 5D).

Similarly, standard morphological analysis showed increased Tb.Th with OA (p<0.001), but the ITS results show increases to only pTb.Th (Fig 5I; p<0.05) and not rTb.Th (Fig 5J). Increased rTb.N (Fig 5H; p<0.001) agreed with the increased mean EF value, indicating a higher presence of rod-like trabeculae in the OA group. These rod-like trabeculae are also significantly longer in the OA specimens (rTb.l, Fig 5L; p<0.01).

### 3.4. Depth-based analysis

**Fig 6:**
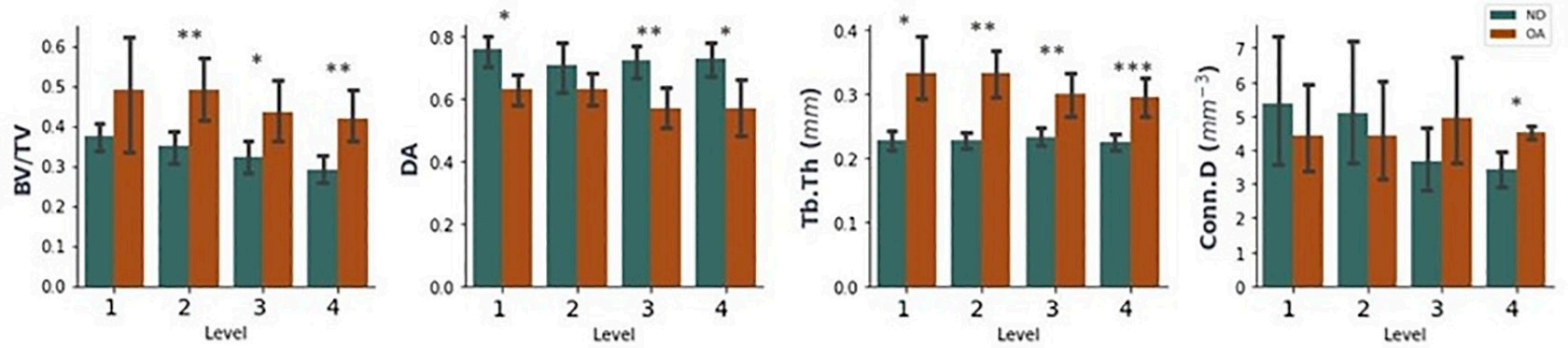
Comparison of AVG (bars) ± SD (error bars) standard morphological properties with increasing depth from the SBP for OA and ND specimens, grouped by level. Levels of significance indicated using: * p<0.05, ** p<0.01, *** p<0.001, ns = no significance.

Standard morphological analysis of each depth-level showed general decreases in BV/TV from Level 1 to 4 in ND tibial specimens (Fig **6**). Comparing ND to OA, higher BV/TV is observed in Level 2 (p<0.01), 3 (p<0.05) and 4 (p<0.01) (Fig 6). DA decreased with OA on Levels 1 (p<0.05), 3 (p<0.01) and 4 (p<0.05) (Fig 6), whilst Tb.Th significantly increases at each level.

**Fig 7:**
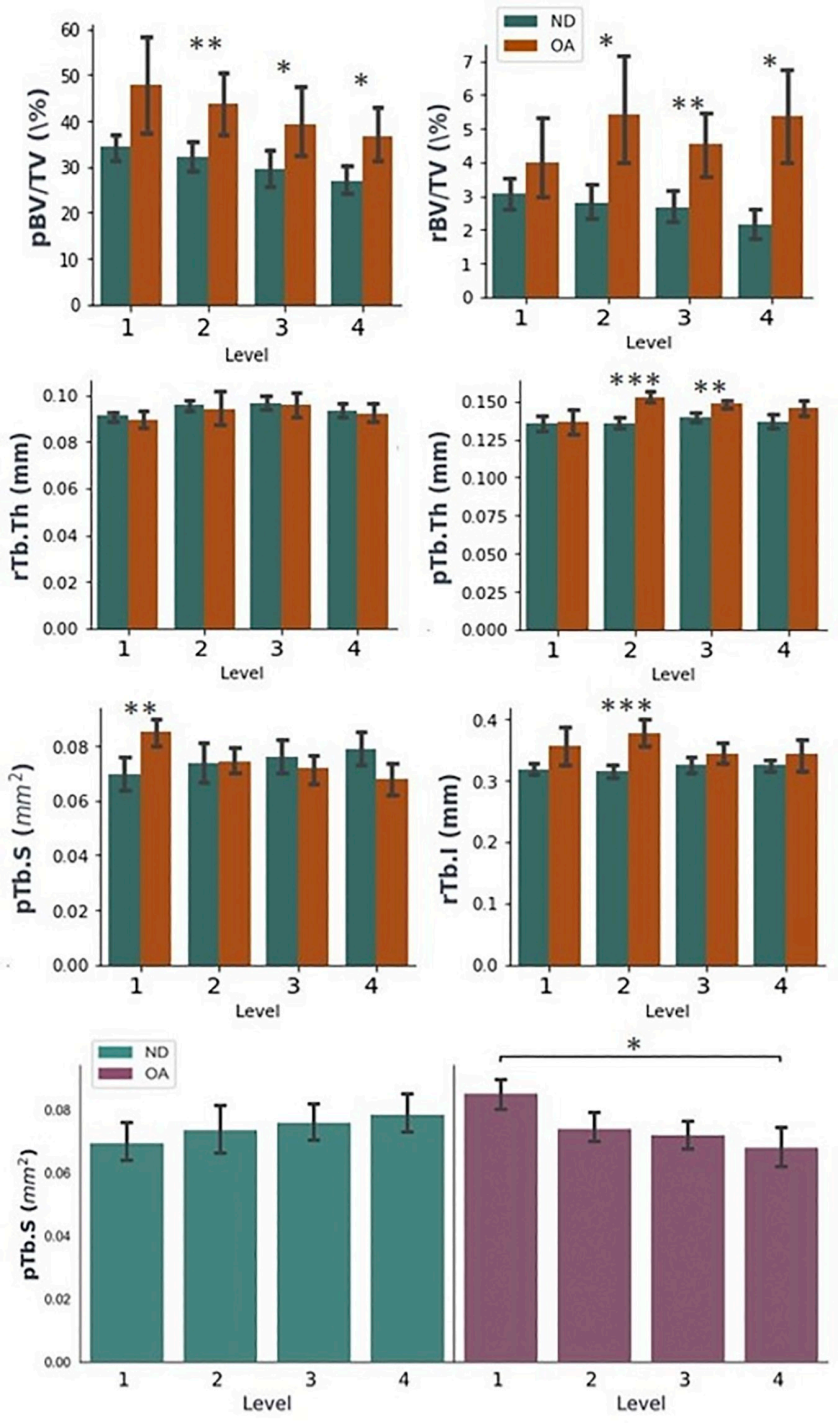
Comparison of AVG (bars) ± SD (error bars) 3D ITS-based morphological properties with increasing depth from the SBP grouped by level (pBV/TV, rBV/TV, rTb.Th, pTb.Th, pTb.S, rTb.l) and showing intra-level variation in properties (pTb.S). Levels of significance indicated using: * p<0.05, ** p<0.01, *** p<0.001, ns = no significance.

The ITS-based morphological analysis highlights more detailed changes to rod and plate characteristics at each level (Table 2). Generally, there was a downward trend in pBV/TV with decreasing depth for both ND and OA samples (Fig 7). However, comparing ND to OA specimens by level showed significant increases to pBV/TV on Levels 2 (p<0.01), 3 (p<0.05) and 4 (p<0.05) (Fig 7). Significant changes to pBV/rBV in Level 2 (p<0.05), Level 3 (p<0.05) and Level 4 (p<0.01) were observed, suggesting a lower plate fraction in lower levels. However, there was a significant increase in pTb.N on Level 4 (p<0.05) with OA. Whilst a significant increase in EF is observed in Level 1 (p<0.05), the ITS results show no significant increases in rTb.N, rBV/TV or rBV/BV at this level (Table 2). rBV/TV values increase at significantly increase at all levels except Level 1 (p<0.05 on Level 2, p<0.01 on Level 3 and p<0.001 on Level 4)(Fig 7). Similarly, increases in rTb.N with OA were observed in the lower levels: 2 (p<0.05), 3 (p<0.05) and 4 (p<0.001) (Table 2). Increases in Level 4 of PPJunc.D (p<0.05) and PR Junc.D Levels 2, 3 and 4 (all p<0.05) were observed in OA samples when compared to ND.

**Table 2:** Comparison of increases (*↑*) or decreases (*↓*) to morphological properties in OA specimens compared to ND controls grouped by depth level. Levels of significance indicated using: * p<0.05, ** p<0.01, *** p<0.001, NS = no significance.

Level	1	2	3	4
**BV/TV**	*↑*ns	*↑* **	↑*	↑**
**DA**	↓*	*↓*ns	↓**	↓*
**Tb.Th**	↑*	↑**	↑**	↑***
**Conn.D**	*↓*ns	*↓*ns	*↑*ns	↑*
**SMI**	*↓*ns	*↓*ns	*↓*ns	*↓*ns
**EF**	↑*	*↑*ns	*↑*ns	*↑*ns
**pBV/TV**	*↑*ns	↑**	↑*	↑*
**rBV/TV**	*↑*ns	↑*	↑**	↑*
**pBV/rBV**	*↑*ns	↓*	↓*	↓**
**aBV/TV**	*↓*ns	*↑*ns	*↑*ns	*↑*ns
**pBV/BV**	*↑*ns	*↓*ns	↓*	↓*
**rBV/BV**	*↓*ns	*↑*ns	↑*	↑*
**pTb.N**	*↑*ns	*↑*ns	*↑*ns	↑*
**rTb.N**	*↑*ns	↑*	↑*	↑***
**pTb.Th**	*↑*ns	↑***	↑**	*↑*ns
**rTb.Th**	*↓*ns	*↓*ns	*↓*ns	*↓*
**pTb.S**	↑**	*↑*ns	*↓*ns	*↓*ns
**rTb.l**	*↑*ns	↑***	*↑*ns	*↑*ns
**RR Junc.D**	*↓*ns	*↑*ns	*↑*ns	*↑*ns
**PP Junc.D**	*↑*ns	*↑*ns	*↑*ns	↑*
**PR Junc.D**	*↑*ns	↑*	↑*	↑*

In OA samples, there was significantly higher plate surface area (pTb.S, p<0.01) towards the SBP, whilst the rod thickness was not observed to be sensitive to depth in either group (Fig 7). Similarly, significantly higher pTb.Th was observed in Levels 2 (p<0.001) and 3 (p<0.01) (Table 2). A significant increase in pTb.S was only observed in Level 1 between OA and controls (p<0.01). Additionally, significantly increased rTb.l was observed on Level 2 of OA specimens compared to controls (p<0.001).

## 4. Discussion

This study evaluated changes to subchondral bone microarchitecture with OA in the distal tibia using standard and ITS-based morphological characterisation. In this way providing an insight to bone remodelling that occurs with the progression of OA and its potential impact on bone strength. Morphological analysis demonstrated an increase in bone volume fraction and trabecular thickness with OA, with a decrease in anisotropy. The OA bone was composed of more rod-like trabeculae and thicker plate-like trabeculae than in ND bone.

In this study, significant increases to BV/TV and Tb.Th were observed in the OA group compared to controls. This observation agrees with other studies that describe increased bone density and trabecular thickness with OA progression in the knee [19, 21, 22, 33–35]; and the hip [36, 37]. It is thought that this occurs as a result of accelerated bone remodelling, resulting in higher apparent bone density to counteract increased loads occuring due to the deterioration of articular cartilage [20]. It was expected that BV/TV would be significantly higher in OA specimens than ND with increasing depth from the SBP, as observed in the OA knee [38–40]. However, the results here show BV/TV increasing with OA at each level, but was no longer significantly different between groups at each level. Similar ankle studies evaluating change in morphological properties with depth have also observed no significant decreases in bone density when analysing *μ*CT images of ND and OA subchondral trabecular bone [41]. However, histological analyses showed significantly higher bone density (p<0.01) in the first 1 mm and between 1–3 mm of late-stage OA tibial bone compared to mild-OA specimens.

Trabecular orientation is known to be an important factor in determining mechanical strength [42–44]. The results in this work show a significant decrease in DA for OA specimens. Studies evaluating mild OA in the knee have observed similar decreases to DA and Conn.D, though non-significant [45]. This suggests that the bone is remodelling to align with the articulating surface, thus reinforcing the joint surface to altered loads, as observed in one study characterising late-stage OA in the proximal tibia [46]. However, whilst an overall increase in aBV/TV is observed with OA, this was found to be non-significant. Similarly, no significant alterations in aBV/TV are observed with depth in the OA group, but significantly higher aBV/TV was observed in the upper levels of ND specimens. The limited sample size of this work may limit the significance observed in these results; a larger dataset could help to identify specific levels of interest where remodelling activity is most pronounced.

An increased mean EF value was observed in the OA group compared to controls, indicating an increase in rod-like trabeculae with OA. Contrastingly, there was a non-significant decrease in SMI value, indicating a more plate-like trabeculae in the OA group. However, SMI does not account for concave surfaces—as featured heavily in trabecular bone- and hence there is a shift towards the use of alternative measures, such as the EF [47, 48]. Though doing so limits the comparisons available to studies that have utilised SMI. Previous studies observed more plate-like trabeculae present in both early [45] and late-stage [19].

OA bone. The results of this study however, show increases to both plate and rod volume fractions, with increased rod tissue fraction and decreased plate tissue fraction in the OA groups compared to controls. Both plate and rod volume fractions significantly increase with OA on Levels 2, 3 and 4, but the results show particularly high rod fraction values for Levels 2 and 4, suggesting unusual remodelling activity at these depths.

An overall plate thickening, increased rod number and rod length was observed in OA specimens. Plate trabecular surface area (pTb.S) was found to be depth-sensitive in the OA group; pTb.S was higher in Level 1 compared to Level 4 and significantly increased with OA in Level 1. Increases to plate thickness and pTb.S suggest an overall thickening of plate-like trabeculae to potentially reinforce their load-bearing capacity [19], particularly in the upper levels nearest the SBP where cartilage loss could be increasing the loads experienced in the bone. The presence of more rod-like trabeculae however, may lead to decreases in mechanical strength [45]. A similar study utilising the same 3D ITS software also found plate thickening, but also a loss of rod-like trabeculae in their OA knee samples [19]. However, this study was analysing much larger volumes (8 mm cuboidal volumes) from across the lateral and medial condyles. Results from one study using smaller cylindrical volumes (5.4 mm and height) compared primary-OA bone to ND controls from the hip. They found increased plate and rod number, but decreased plate and rod thickness, plate surface area and rod length [24]. It is therefore difficult to draw comparisons to other lower-limb joints, due to variations in sample size and geometry, but also due to the differences in joint biomechanics which may alter OA progression.

To the authors knowledge, few studies have reported these metrics for the osteoarthritic ankle. Obtaining osteoarthritic ankle samples is difficult, namely as ankle arthroplasty and arthrodesis surgeries are less common than those conducted in the hip and knee NJR (2019). The differences in sample size, location and even the type of OA present (ankle OA is predominantly post-traumatic, though this cannot be confirmed for the specimens used in this study) will affect the strength of the conclusions drawn from these results. As mentioned previously, it is also difficult to draw comparisons to the few lower-limb studies available in the literature, to begin to understand the impact of OA on ankle subchondral bone. Furthermore, unlike other lower-limb joints, it is challenging to source donor-matched or age-matched OA and ND ankle samples. In this case, the OA specimens were from older donors compared to the cadaveric tissue. Moreover, it was difficult to correspond the state of the cartilage in the OA samples, as the specimens were taken from multiple locations during joint space preparation, and therefore may not necessarily display fully degraded cartilage as they may not have been removed from the primary loading zone. Hence, there is unavoidable regional variation in the OA sample group, which somewhat limits the comparisons to the ND control specimens, since they were retrieved from the main articulating region of the tibial plafond. Furthermore, to maximise available cadaveric and OA tissues, multiple samples were obtained from one donor where possible. Whilst these are not true biological replicates, and therefore can not be treated as completely independent, they have provided critical data on the micro-scale variation to the bone morphology in the ankle.

Exploring the link between mechanical and structural properties can aid predicting surgical outcomes [49]. Higher bone density and trabecular thickness may be beneficial for the performance of arthroplasty and arthrodesis by improving fixation into the bone, reducing the risk of loosening [50].

## 5. Conclusion

This study has demonstrated that significant changes to ankle subchondral bone are observed in late-stage OA, such as increased BV/TV and Tb.Th. Significant alterations to trabecular morphology were observed and believed to be the result of accelerated remodelling. Hence, testing interventions on non-diseased bone is not sufficient, particularly as some properties were depth-dependent in osteoarthritic bone. Future studies also could look to analyse cartilage thickness and correlate these to changes in trabecular bone morphology. In this way, the response of subchondral trabecular bone to the progression of OA can be speculated and changes to biomechanical strength inferred.
